# A Novel Posterior Implant System for Sacroiliac Joint Fusion: Preliminary Cadaveric and Clinical Results

**DOI:** 10.7759/cureus.89633

**Published:** 2025-08-08

**Authors:** James North, Connor Huxman, Joshua Tandio, Oluwatodimu R Raji, Jeremi M Leasure, Jonathan A Hyde, Andrew Hyde, Thomas P Hedman, Adam Rogers

**Affiliations:** 1 Pain Management, Carolinas Pain Institute, Winston Salem, USA; 2 Research, Spinal Simplicity, LLC, Overland Park, USA; 3 Research, Medical Device Development, San Francisco, USA; 4 Orthopaedic and Spinal Surgery, Miami Spine Specialists, Miami Beach, USA; 5 Medicine, St. George’s University School of Medicine, St. George's, GRD; 6 Biomedical Engineering, University of Kentucky, Lexington, USA

**Keywords:** biomechanics, degenerative sacroiliitis, fusion device, patient-reported outcome measures, posterior intra-articular technique, sacroiliac joint dysfunction, sacroiliac joint fixation

## Abstract

Background

Sacroiliac joint fusion is performed to stabilize and fuse the joint in patients with degenerative sacroiliitis and joint dysfunction. While several posterior techniques and implants exist as alternatives to lateral approaches, biomechanical and clinical performance data for these systems used as standalone remains limited. This article provides a preliminary cadaveric and clinical assessment of a novel posterior intra-articular sacroiliac fusion implant system.

Methods

A cadaveric biomechanical evaluation was conducted comparing the posterior implant to a posterolateral transiliac implant, evaluating each device's ability to reduce flexion/extension motion after initial fixation and fatigue loading when used as standalone fixation. Anatomical safety analysis was performed on each implant’s post-fixation position, measuring minimum distances to critical SI landmarks on computed tomography (CT) scans. A retrospective clinical evaluation was also conducted, assessing early patient-reported outcomes for patients treated with the posterior device as standalone.

Results

Biomechanical stability in flexion/extension loading was greater with the posterior implant than the posterolateral implant, both post-fixation (31% motion reduction posterior; 4% increase posterolateral) and after fatigue loading (28% motion reduction posterior; 5% increase posterolateral). Strong effect sizes between implant groups were computed post-fixation (d = 1.92, p = 0.201) and post-fatigue (d = 1.63, p = 0.207), although these differences were not statistically significant with the limited sample size and inter-specimen variability. Compared to the posterolateral implants, the posterior implants were positioned approximately five times further from the sacral foramen (+13.6 mm, p = 0.020) and four times further from the anterior sacral border (+18.5 mm, p = 0.104). Clinical data on 10 patients treated with 13 standalone posterior implants found statistically significant improvements for mean Numerical Rating Scale (NRS) pain scores (4.7 points, 59% improvement, p = 0.014) and Oswestry Disability Index (ODI) scores (10 points, p = 0.029) at an average follow-up of 97 days. All domains of the Patient-Reported Outcomes Measurement Information System (PROMIS-29) assessment also improved (17% mean improvement), with most (4/7) domains showing statistically significant improvement. No device-related adverse events, removals, revisions, or follow-up procedures were reported.

Conclusion

These preliminary results suggest potential advantages in stability and safety for the novel posterior implant used as a standalone when compared to standalone posterolateral fusion. Early clinical data are promising, showing timely, statistically significant improvements in patient-reported outcomes. Additional biomechanical data and long-term prospective clinical data, including radiographic fusion evaluation, will be necessary to validate these results and further evaluate device performance.

## Introduction

Sacroiliac (SI) joint disruption is recognized as a significant cause of low back pain [1-6]. Fusion of the SI joint is indicated in patients with significant and persistent joint pain and degenerative pathology for whom conservative treatment has not provided relief. Several surgical approaches exist for the placement of SI fusion devices, including transiliac lateral approaches, transiliac posterolateral approaches, and posterior intra-articular approaches. While implant designs and surgical techniques are considerably varied today, these devices share a similar purpose of stabilizing the joint and facilitating bony fusion between the sacrum and ilium to reduce or eliminate joint motions and stem further degeneration and pain.

Posterior intra-articular SI fusion devices are used to treat SI joint instability and offer a potentially less invasive surgery by avoiding the neurovascular bundle, which is associated with transiliac approaches [7-10]. Posterior implants inserted directly into the SI joint may reduce certain intraoperative risks such as implant breach of the neuroforamen or anterior sacral cortex [11], though safety data comparing techniques remains limited. Posterior SI implants have also been used by some as a salvage procedure for pseudarthrosis following previous lateral SI joint fixation [12]. Many intra-articular devices require use in a hybrid construct alongside a lateral implant, and the biomechanical performance of standalone intra-articular fixation has not been thoroughly investigated.

When evaluating new SI joint fusion technologies, it is critical to assess their ability to stabilize SI joint motions when subjected to the various biomechanical loading modes experienced in activities of daily living. Previous works by Jeong et al. [13], Sayed et al. [14], Raji et al. [15], and others [16-18] have developed and utilized cadaveric test methods incorporating pure moment loading, static and fatigue regimens, soft tissue resection techniques, and motion capture methods for quantifying stabilization pre- and post-implantation. Given the variation that exists between individual cadaveric specimens, recent emphasis has been placed on the importance of concurrent controls in these investigations. This approach is utilized in this study and involves the placement of comparator devices in contralateral SI joints to control for bone quality and anatomical variation between specimens.

In addition to biomechanical performance, assessment of clinical outcomes is necessary to capture pain and functional improvements as well as real-world safety and performance data. This study introduces a novel posterior intra-articular SI fusion device placed directly into the joint space. The primary objective of this study was to evaluate the biomechanical stability and preliminary clinical outcomes of a novel posterior intra-articular SI joint fusion device compared to a commonly used posterolateral device. The secondary objective was to assess clinical safety and cadaveric implant positioning, including the distances to critical anatomical landmarks. 

## Materials and methods

Novel posterior intra-articular implant

**Figure 1:**
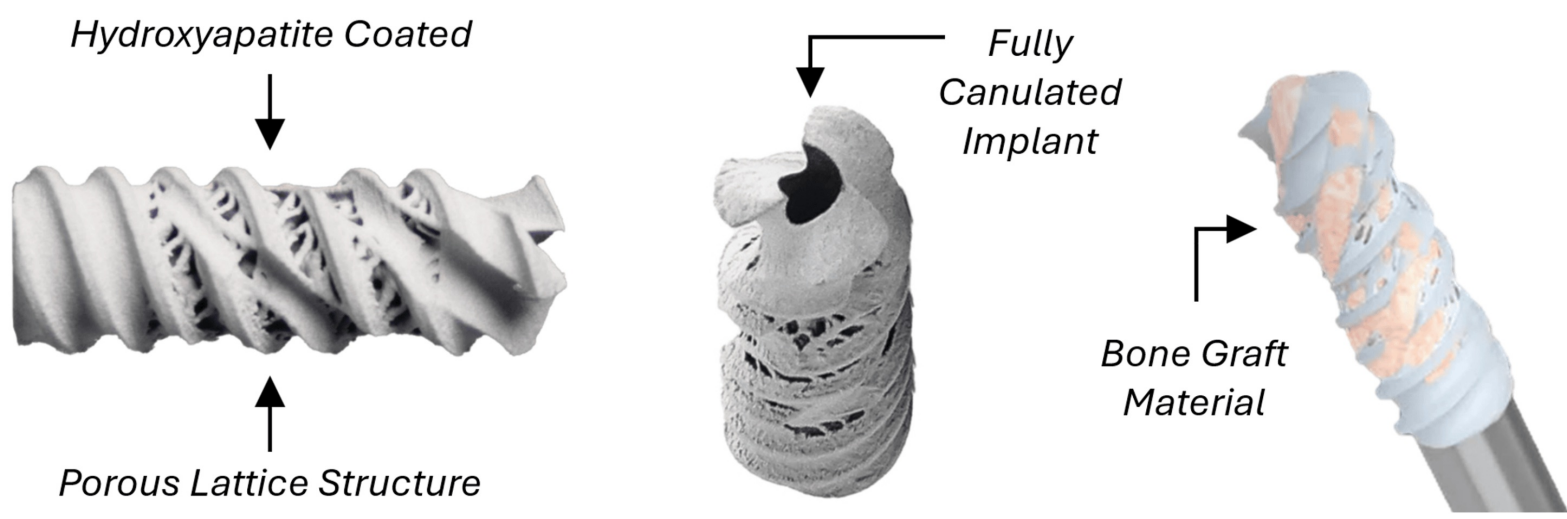
The Patriot-SI™ posterior sacroiliac fusion implant (Spinal Simplicity, LLC, Overland Park, KS) Image contents adapted from product technique guides with permission from Spinal Simplicity. Product indications available at [19].

This study investigates the Patriot-SI™ (Spinal Simplicity, LLC, Overland Park, KS), a minimally invasive intra-articular implant intended for the stabilization and fusion of the SI joint. The device, shown in Figure 1, is placed through a posterior approach in the articular portion of the joint (in line with the joint), piercing the cortices of both the sacrum and the ilium as the threads engage the bone and provide fixation. The Patriot-SI™ has additively manufactured lattice structures and fenestrations and is made from titanium alloy (Ti6Al4V) with a hydroxyapatite coating to improve stability and facilitate fusion of the joint. The implant has a tri-lead thread design, is 10 mm in diameter, and is fully canulated. The Patriot-SI™ system is the only device of its kind that can be precisely guided into place within the joint using a guidewire through the implant. Under fluoroscopic guidance, the device is placed through a minimally invasive posterior approach with proper implant placement being anterior to the posterior sacral cortical outline, superior to the inferior aspect of the ilium, and with the distal tip as close as possible to the anterior sacral cortical wall.

Biomechanical evaluation

Cadaveric biomechanical testing was conducted comparing the Patriot-SI™ posterior device and a commonly used posterolateral fusion device, the RIALTO™ (Medtronic, Minneapolis, MN), each used as standalone fixation. Devices were implanted randomly into either left or right SI joints of three cadaver pelvises using the same protocol as used previously [13-15], which involved sex stratification, ensuring side assignments were equally distributed between both implants for specimens of the same sex. The mean age of specimens was 56 ± 11, BMI was 27.5 ± 2.4, bone quality (L4 t-scores) was -1.0 ± 0.4, and the sex distribution was 2:1 male:female. All specimens were screened to exclude those with bony bridging, SI joint disease, or poor bone quality. Causes of death for the three donors were metastatic neuroendocrine cancer, end-stage renal disease, and multiple causes, including hypertensive cardiovascular disease, diabetes, toxic effects of methamphetamine, and environmental heat stress. All device placements were performed per each manufacturer’s surgical technique and were reviewed and approved by a board-certified orthopedic surgeon. Pure-moment flexion/extension bending was applied to specimens from 0 to 7.5 Nm in the following three configurations: i) intact, ii) destabilized (resection of the sacrotuberous, sacrospinous, and iliolumbar ligaments, and the pubic symphysis), and iii) post-fixation conditions [15] (mechanical test frame MTS 858 MiniBionix II, MTS Systems Corp, Eden Prairie, MN, USA). Subsequently, constructs were subjected to 5,000 cycles (1 Hz, sinusoidal waveform) of fatigue loading from 0 to 5.625 Nm in extension [18], followed by a final round of pure moment loading to assess durability of fixation. For all test configurations, motion tracking of optical markers (Optotrak, Northern Digital, Waterloo, Ontario, Canada) was used to assess relative motions between the sacrum and ilium [13-15].

A safety assessment comparing the positions of the two implants was also conducted. Measurements were taken from post-fixation computed tomography (CT) scans (Radiant DICOM Viewer, Medixant Pozna, Poland) to determine the minimum distances from each implant to the sacral foramen and to the anterior border of the sacrum. The measurement plane was selected by aligning one CT axis with the principal axis of the implant and rotating the CT around this axis to identify the shortest distances to critical structures. These distances were defined as the shortest distance from any location on the implant (typically the side of the implant) to any location on the foraminal border, or the distance from the nose of the implant to the anterior cortical wall, bounded by the trajectory of the implant. A single rater from the Medical Device Development team collected measurements, which have been validated as having an intraclass correlation coefficient (ICC) as low as 0.83.

Given the small sample size in this analysis and the inherent variability associated with cadaveric specimens, this study was not powered to detect specific differences between groups, but rather to uncover potential trends that can be further explored and validated with additional testing. Statistical analysis of these initial findings was performed in R (RStudio, Posit Software, PBC, Boston, MA, USA). Range of motion reductions were compared across implants and after fixation and fatigue conditions with a one-way analysis of variance (ANOVA). Pairwise comparisons were performed with Tukey’s honest significant difference (HSD) tests (α = 0.05). Implant position measurements were compared with two-tailed unpaired t-tests (α = 0.05). Normality was verified for all analyses (Shapiro-Wilk tests), and effect sizes were computed (Cohen’s d). All values are expressed as mean ± standard deviation.

Clinical evaluation of standalone use

Clinical data from the first 10 patients treated with the Patriot-SI™ device as standalone fixation by the first author were retrospectively reviewed. This sample size was selected to obtain timely clinical safety and performance data on the standalone use of the device when used per standard of care in the commercial environment. Assessment of this early cohort of the first 10 patients will allow for the identification of potential trends, informing future prospective studies involving larger sample sizes and follow-up timeframes. This retrospective analysis identified patients for whom selection, treatment, and follow-up had already occurred in the commercial environment. Clinical selection of appropriate patients to receive the Patriot-SI™ system was performed by the site per standard of care and is detailed in the following subsection. The inclusion criteria for this study were specified to include patients in the retrospective analysis if they had (1) previously received one or more standalone Patriot-SI™ implants and (2) previously completed outcome assessments at both baseline and follow-up. These outcome assessments included the Patient-Reported Outcomes Measurement Information System (PROMIS-29), Numerical Rating Scale (NRS) pain scores, and Oswestry Disability Index (ODI) scores. The timeframe for patients completing follow-up questionnaires was approximately three months, as is standardly performed by the treating physician’s practice. Given the real-world, retrospective nature of this data, exact follow-up time varied across patients, as is reported in the results. Any adverse events, revisions, removal procedures, or follow-up procedures at the index level were recorded. 

Statistical analysis of clinical data was performed in R. Following Shapiro-Wilk normality tests, outcome measure follow-up and baseline scores were compared with paired two-tailed t-tests or Wilcoxon signed-rank tests. To investigate whether follow-up time was correlated with clinical outcomes, unpaired t-tests or Mann-Whitney U-tests were run between equally sized subcohorts (n = 5) of patients with the five shortest and longest follow-up times, comparing scores for NRS, ODI, and all PROMIS-29 domains. Considering the preliminary nature of this clinical investigation, which is not intended to yield definitive conclusions but rather to uncover findings warranting further investigation, as well as the use of non-parametric tests across a reasonable number of outcomes, no additional multiplicity adjustments were applied, and α=0.05 was used for statistical significance. To further contextualize clinical results, the number of patients achieving a minimum clinically important difference (MCID) for each outcome measure is reported. MCID thresholds used were based on commonly reported values in the literature: ≥5 points for all PROMIS-29 domains, which has been reported as a reasonable threshold across domains for low back pain [20], ≥2 points for NRS pain [21], and ≥10 points for ODI [22,23].

Patient selection

This retrospective study evaluated patients for whom treatment and follow-up were already completed in the commercial environment; thus, patient selection was performed per standard of care at the treating physician’s site outside the scope of this study. This patient selection assessment involved standard screening of patient symptoms, previous attempted treatments, presence of comorbidities, and other medical information. Potential candidates for the Patriot-SI™ fusion system are typically those with SI joint pain, failure of conservative care (≥ 6 months of physical therapy, medications, behavior/exercise modifications, and/or injections), and ≥75% pain relief following fluoroscopy-guided SI joint injections. Diagnostic imaging, physical examination, and joint injections are commonly used to isolate the SI joint as the patient’s primary pain generator.

## Results

Biomechanical results

**Figure 2:**
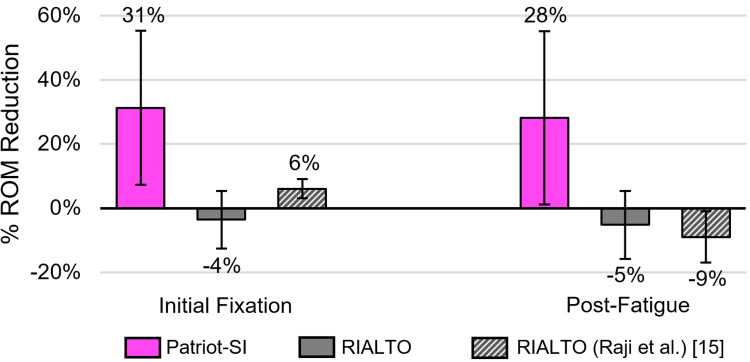
Percent reduction in flexion/extension range of motion (ROM) after initial fixation (left) and post-fatigue 5,000 cycles (right). RIALTO™ data from Raji et al. [15] after 18,500 cycles in the same test setup is also shown. Percent reductions had a large effect size between implants but were not statistically significant for initial fixation (d = 1.92, p = 0.201) and post-fatigue (d = 1.63, p = 0.227).

Figure 2 shows the flexion/extension range of motion (ROM) reductions for each device. The Patriot-SI™ reduced (stabilized) flexion/extension motion from the destabilized value of 3.2 ± 1.5˚ to 2.1 ± 0.7˚ for initial fixation (31% ± 24% reduction) and 2.3 ± 0.9˚post-fatigue (28% ± 27% reduction). On average, RIALTO™ increased (did not stabilize) motion compared to the destabilized value of 3.2 ± 1.9˚ for both the initial fixation condition (3.4 ± 2.2˚; 4% ± 9% increase) and post-fatigue condition (3.4 ± 2.1˚; 5% ± 11% increase). After verifying data normality, the effect size between implant groups was computed as large for both initial fixation (d = 1.92) and after fatigue loading (d = 1.63). The differences between groups, however, were not statistically significant (fixation: p = 0.201; fatigue: p = 0.227) due to small sample size and variance across specimens.

**Figure 3:**
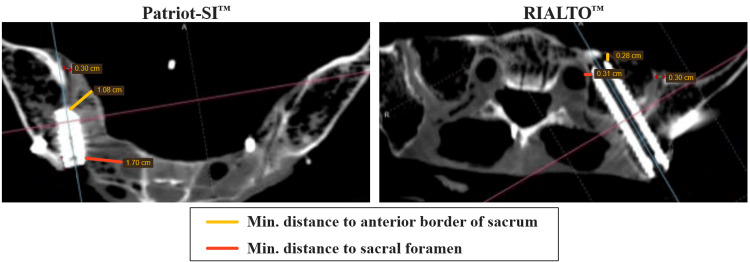
Representative post-fixation CT scans and safety measurements for each implant in a single specimen Measurements highlighted in yellow and red for minimum distances to anterior border of sacrum and sacral foramen, respectively. Text overlayed on initial labels for legibility. Anterior joint width measurements after fixation also shown for reference.

As shown in Table 1, the safety analysis revealed that compared to the RIALTO™ implant, the Patriot-SI™ implant position was approximately four times further from the anterior sacral border (24.2 ± 11.6 mm versus 5.7± 2.6 mm; p = 0.104, t = 2.690) and five times further from the sacral foramen (16.9 ± 4.1 mm versus 3.3 ± 1.4 mm; p = 0.020, t = 5.417). This increased safety margin with Patriot-SI™ was statistically significant for the sacral foramen, and the effect size between implant groups was computed as large for both distances (anterior sacral border: d = 2.20, sacral foramen: d = 4.42). Figure 3 includes representative fixation scans of each device implanted into a single specimen, with safety measurements to the critical landmarks annotated.

**Table 1:** Implant position assessment. *Indicates statistically significant difference (α = 0.05). The ratio reported represents the increase in distance with Patriot-SI™ compared to RIALTO (ratio of means for both measurements).

	Patriot-SI^™^	RIALTO^™^	Ratio (Patriot-SI^™^ : RIALTO^™^)	p-value
Min. distance to anterior border of sacrum	24.2 ± 11.6 mm	5.7 ± 2.6 mm	4.2 : 1	0.104
Min. distance to sacral foramen	16.9 ± 4.1 mm	3.3 ± 1.4 mm	5.1 : 1	0.020*

Clinical results

Ten patients (13 implantations) treated with the Patriot-SI™ device as standalone fixation by the first author were included for retrospective analysis. The mean age was 71.3 ± 11.6 years (range 48-86), and 60% of patients were female. Seven devices were implanted in the left SI joint, and six were implanted in the right SI joint. All patients completed the outcome measure assessments at baseline and at follow-up, after all implantations, which averaged 97 ± 41 days (range 35-145) after surgery. Three patients received two Patriot-SI™ implants (one on each side) with an average time between implantations of 12 ± 9.2 days (range 2-20).

**Figure 4:**
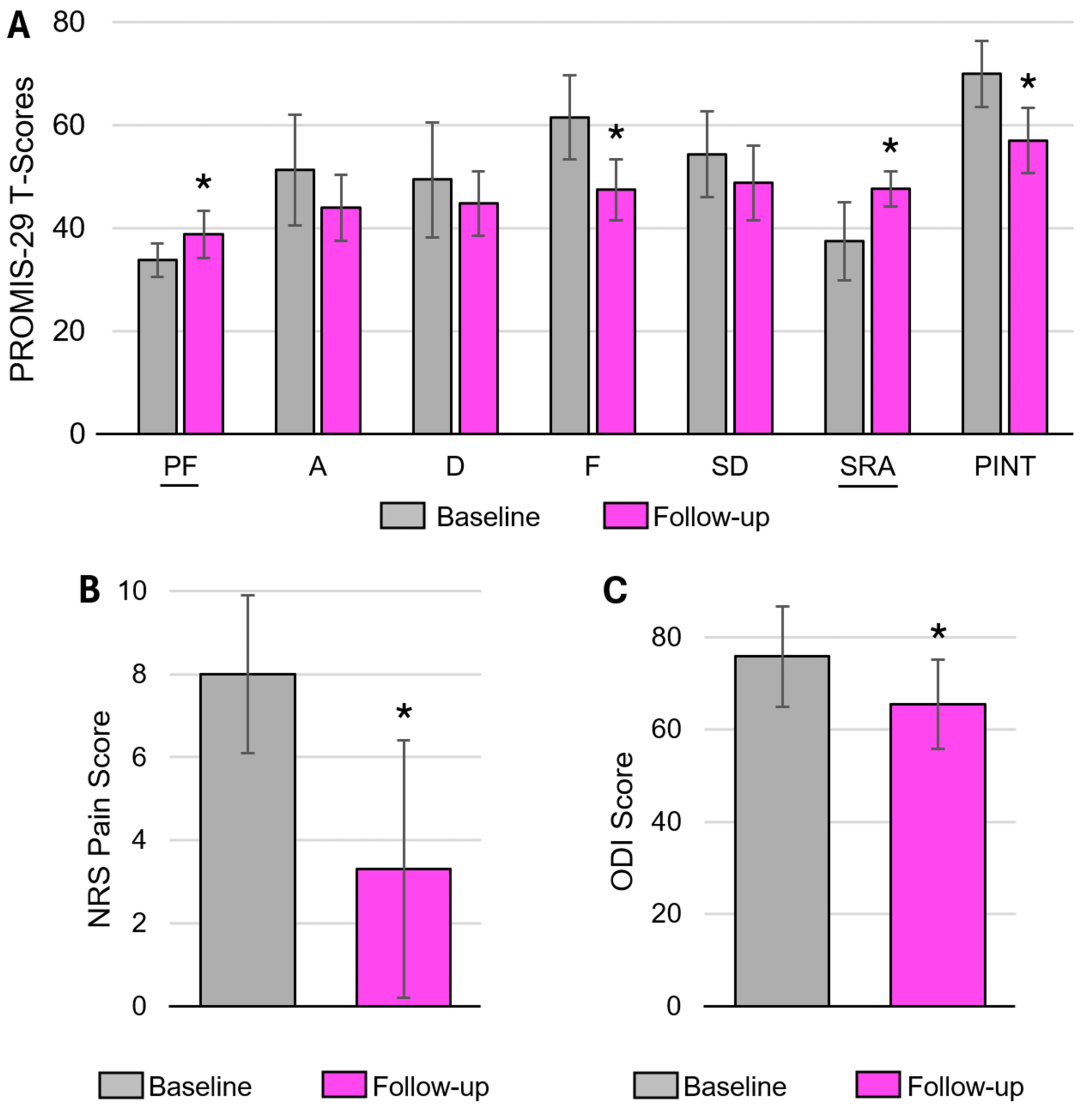
Baseline and follow-up scores for (A) PROMIS-29, (B) NRS Pain, and (C) ODI. *Indicates statistically significant improvement from baseline (p < 0.05). Note: PROMIS-29 domains underlined represent those for which increases indicate improvement: PF (physical function) and SRA (social roles and activities). For all other domains, decreases indicate improvement: A (anxiety), D (depression), F (fatigue), SD (sleep disturbance), and PINT (pain interference).

Figure 4A and Table 2 show improvements in all PROMIS-29 domains from baseline to follow-up. Mean improvement in PROMIS-29 T-scores was 17% ± 6% across all domains, which included physical function (PF), anxiety (A), depression (D), fatigue (F), sleep disturbance (SD), social roles and activities (SRA), and pain interference (PINT). Table 2 shows mean scores and improvements, computed confidence intervals (CI), and test statistics. Statistically significant improvements were found for the following domains: PF (5.0 points, p = 0.002), F (14 points, p = 0.004), SRA (10.2 points, p = 0.004), and PINT (13 points, p = 0.002). Improvements were also observed but not statistically significant for A (7.3 points, p = 0.082), D (4.6 points, p = 0.089), and SD (5.6 points, p = 0.345) domains. The largest improvement was in F (22.8%) while the smallest was in D (9.4%). On average across all domains, more than half (57%) of patients achieved MCID thresholds, with functional and pain-related domains showing stronger improvements (60% for physical function, 67% for fatigue, and 88% for pain intensity).

**Table 2:** Clinical outcome measure scores for PROMIS-29 domains, NRS pain, and ODI. Bolded rows indicate statistically significant improvements from baseline (p < 0.05). Baseline, follow-up, and mean improvement columns reported in points. Confidence intervals (CI) for mean improvement are reported at the 95% level or the highest level that can be computed for non-parametric tests with a limited sample size. Note: PF (physical function) and SRA (social roles and activities) PROMIS-29 domains show improvement when scores increase. For all other domains, decreases indicate improvement: A (anxiety), D (depression), F (fatigue), SD (sleep disturbance), and PINT (pain interference).

Outcome Measure	Baseline	Follow-up	Mean improvement	p value	Test statistic	% achieving MCID
PROMIS-29						
PF	33.8 ± 3.2	38.8 ± 4.7	5.0 (95% CI: 2.27 – 7.61)	0.002	t=4.192	60% (6/10)
A	51.3 ± 10.7	44.0 ± 6.9	7.3 (90% CI: -7.70 – 27.00)	0.104	z=1.62	40% (4/10)
D	49.4 ± 10.1	44.8 ± 6.3	4.6 (60% CI: 10.80 – 17.90)	0.181	z=1.12	30% (3/10)
F	61.5 ± 8.9	47.5 ± 6.6	14.0 (95% CI: 5.77 – 21.65)	0.004	t=3.982	67% (6/9)
SD	54.4 ± 9.2	48.8 ± 5.6	5.6 (95% CI: -5.16 – 12.89)	0.345	t=1.012	38% (3/8)
SRA	37.4 ± 7.4	47.6 ± 3.2	10.2 (95% CI: 4.75 – 14.70)	0.022	z=3.13	75% (6/8)
PINT	70.0 ± 6.2	57.0 ± 7.0	13.0 (95% CI: 8.30 – 22.50)	0.022	z=3.19	88% (8/9)
Numerical Rating Scale (NRS) Pain						
NRS	8.0 ± 1.9	3.3 ± 3.1	4.7 (95% CI: 3.5 – 8.0)	0.014	z=2.94	80% (8/10)
Oswestry Disability Index (ODI)						
ODI	75.8 ± 10.9	65.5 ± 9.7	10.3 (95% CI: 1.3 – 19.3)	0.029	t=2.598	40% (4/10)

Figure 4B shows statistically significant improvements in NRS pain scores. A mean improvement of 59% in pain scores was reported (4.7 points, p = 0.014, z = 2.94). ODI scores also showed statistically significant improvements (Figure 4C) from 75.8 ± 10.9 at baseline to 65.5 ± 9.7 at follow-up, representing a 10.3-point (14%) improvement (p = 0.029, t = 2.598). Across the 10 patients, 80% achieved MCID for NRS pain and 40% achieved MCID for ODI. These observed clinical improvements were not found to be correlated with exact follow-up time. The five patients with the shortest follow-up and the five patients with the longest follow-up had no statistically significant differences in score changes across NRS, ODI, and all PROMIS-29 domains.

**Figure 5:**
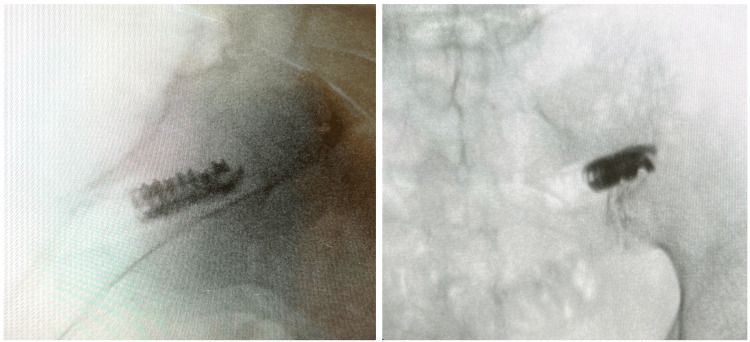
Intraoperative radiographs showing placement of the Patriot-SI™ in a 66-year-old female patient

None of the patients reported a device-related adverse event, no patient had a revision or removal procedure, and no patients had a follow-up procedure at the index level. Figure 5 shows intraoperative radiographs, highlighting the anatomical position of the implanted Patriot-SI™ device. This 66-year-old patient treated with the Patriot-SI™ had a five-year history of chronic right-sided back and buttock pain, consistent with sacroiliac joint pain, and was diagnosed with sacroiliac joint dysfunction. Prior treatments included nonsteroidals, bisphosphonate therapy, intermittent SI injections, and lateral branch radiofrequency ablation.

## Discussion

This study included both a preliminary cadaveric and clinical assessment of a novel posterior intra-articular SI fusion implant used as a standalone. The results suggest that the posterior implant can provide significant and timely clinical improvements in patients treated with the standalone implant, and that the device may provide advantages in stability and safety when compared to a posterolateral implant.

Preliminary investigations of new spinal and orthopedic technologies can provide timely assessments of safety and performance and identify potential advantages, trends, and inform future research questions. By combining both cadaveric and clinical evidence, this study provides an initial, multi-modal evaluation of the subject posterior SI fusion device’s performance. The preliminary cadaveric biomechanical assessment found that fixation of destabilized joints with the posterior implant reduced motion on average by 31% initially and 28% after fatigue loading, while the posterolateral implant did not reduce motion initially (4% increase) or after fatigue loading (5% increase). These results are comparable to those of Raji et al. [15], who found a 6% reduction initially and a 9% increase after fatigue loading for the same posterolateral implant in the same test setup, subjected to nearly four times greater fatigue cycles. The present results favor Patriot-SI™ with strong effect sizes computed both after fixation and after fatigue (d = 1.92 and 1.63, respectively), although statistical significance was not achieved with this limited sample size (p = 0.201 and 0.227, respectively), and thus definitive conclusions cannot be made. While not statistically significant, this trend toward greater initial and post-fatigue stability with Patriot-SI™ could translate to greater potential for joint fusion and may reduce the risk of implant subsidence and migration during the critical months following surgery as fusion takes place. However, this analysis was a worst-case biomechanical comparison involving one posterior versus one posterolateral implant, whereas in clinical practice, the use of two posterolateral implants is commonly performed. The present test design has become a standard approach used for benchmarking of devices to support market clearance in the U.S., which allows for direct comparisons to other studies, although clinical interpretation must consider these differences. This analysis also involved a small number of specimens with considerable variability in anatomy and bone quality, as is common with cadaveric testing. Additional validation is needed with adequate statistical power before generalizing these results. Repeat testing conducted on more specimens will determine whether motion differences between groups are statistically significant, and testing under additional loading modes (lateral bending, axial rotation, axial compression) will provide a more comprehensive assessment of biomechanical stability. 

The biomechanical safety analysis revealed an important difference between the anatomical positions of the posterior and posterolateral implants. The posterior implant was positioned approximately four to five times further from the anterior sacral border and sacral foramen than the posterolateral implant. Strong effect sizes were observed for both measurements (d = 2.20 and 4.42, respectively), and the difference was statistically significant for the sacral foraminal distance (p = 0.020) but not for the anterior sacral border (p = 0.104). Considering the interspecimen variation, evaluation in additional specimens is needed to further characterize the statistical differences between implants. However, this strong trend toward an increased margin of safety for the posterior implant may help avoid breaching the neuroforamen or anterior sacral cortex, which have been documented as risks associated with various SI trajectories and implants [11,24,25]. Specifically, the majority of SI fusion complications are due to improper implant placement [24], and the most common adverse event attributed to malposition is nerve root impingement, as reported in a systematic review by Shamrock et al. [25]. Most notably, it is encouraging in the current study that an overall smaller implant may improve both the biomechanical stability and the safety profile by occupying less space and minimizing these critical distances. It should be noted, however, that these anatomical safety measurements are from cadaveric experiments and have not yet been validated in larger clinical trials.

The clinical data presented here on the subject posterior SI device are promising, providing early insight into pain, functional, and safety outcomes. All three outcome metrics evaluated, PROMIS-29, NRS pain, and ODI scores, demonstrated improvements versus baseline, and there were no reoperations, removals, follow-up procedures, or device-related adverse events. This study’s low complication rate (0%) and mean improvements in pain (59%), disability (10 points), and PROMIS-29 (17%) scores appear consistent with other studies evaluating posterior SI fusion at early time points. Other studies have reported complication rates of 0-4.3%, mean pain reductions of 47-73%, disability improvements of five to 24 points, and PROMIS-29 score improvements of 18% [26-29], although some differences exist in methodology, including the use of Visual Analogue Scale (VAS) pain instead of NRS. It should be noted that follow-up time varied considerably across patients, which, while common for retrospective analyses, is a limitation of this study. The present data is also preliminary and includes the extent to which it is collected per standard of care at a single site for a small number of patients and implantations. The single-physician, non-controlled design of this study limits its generalizability. While the limited sample size and timeframe allowed for timely assessment of a novel device, the data are not sufficient to perform subgroup analyses to identify additional trends or better inform future patient selection. Physician-initiated studies across multiple sites with additional and longer follow-up time points would help reduce bias. However, the results are encouraging and warrant further evaluation, which may include prospective analyses for a larger number of patients at longer follow-ups. Fusion was also not evaluated in this retrospective study, but is planned for future radiographic investigations.

## Conclusions

This study evaluated both the biomechanical and clinical performance of a novel posterior intra-articular SI fusion device. These preliminary findings demonstrate that when used as a standalone fixation device, the Patriot-SI™ implant may provide increased flexion/extension stability compared to a standalone posterolateral SI fusion device, can increase the safety margin to critical SI anatomical landmarks, and can provide timely, statistically significant improvements in patient-reported outcomes. Given the documented complications involving improper implant placement and breaching of the sacral foramen and anterior sacral cortex, this preliminary cadaveric data suggest that the subject posterior SI implant represents a new and potentially advantageous approach lying further from these landmarks while providing comparable or improved flexion/extension stability. Additional cadaveric biomechanical testing and clinical radiographic evaluation of fusion will be needed to validate and expand upon these results, which will help inform clinical decision-making around implant selection.
